# Cross-sectional Kelvin probe force microscopy on III–V epitaxial multilayer stacks: challenges and perspectives

**DOI:** 10.3762/bjnano.14.59

**Published:** 2023-06-14

**Authors:** Mattia da Lisca, José Alvarez, James P Connolly, Nicolas Vaissiere, Karim Mekhazni, Jean Decobert, Jean-Paul Kleider

**Affiliations:** 1 Institut Photovoltaïque d'Ile de France, 30 Route Départementale 128, 91120, Palaiseau, France; 2 Université Paris-Saclay, CentraleSupélec, CNRS, Laboratoire de Génie Electrique et Electronique de Paris, 91192, Gif-sur-Yvette, France; 3 Sorbonne Université CNRS, Laboratoire de Génie Electrique et Electronique de Paris, 75252, Paris, France; 4 III-V Lab, 1 Avenue Augustin Fresnel, 97167 Palaiseau, France

**Keywords:** FM-KPFM, frequency-modulated Kelvin probe force microscopy, III–V multilayer stack, Kelvin probe modelling, KP modelling, SPV, surface photovoltage

## Abstract

Multilayer III–V-based solar cells are complex devices consisting of many layers and interfaces. The study and the comprehension of the mechanisms that take place at the interfaces is crucial for efficiency improvement. In this work, we apply frequency-modulated Kelvin probe force microscopy under ambient conditions to investigate the capability of this technique for the analysis of an InP/GaInAs(P) multilayer stack. KPFM reveals a strong dependence on the local doping concentration, allowing for the detection of the surface potential of layers with a resolution as low as 20 nm. The analysis of the surface potential allowed for the identification of space charge regions and, thus, the presence of several junctions along the stack. Furthermore, a contrast enhancement in the surface potential image was observed when KPFM was performed under illumination, which is analysed in terms of the reduction of surface band bending induced by surface defects by photogenerated carrier distributions. The analysis of the KPFM data was assisted by means of theoretical modelling simulating the energy bands profile and KPFM measurements.

## Introduction

The development of photovoltaic (PV) technologies has progressed significantly over the past twenty years as a result of considerable advancements in solar cell device engineering and material science. As a consequence, solar cells have turned into complex structures containing numerous layers and interfaces [1]. The capability to conduct local investigations at the nanoscale level that provide information on the electrical properties of materials and along physical interfaces is becoming crucial for solar photovoltaic device efficiency improvement [2].

Electrical measurements based on scanning probe microscopy (SPM) allow for the analysis of two-dimensional (2D) features at the surface and along a physical cross section of nanoscale semiconductor structures. Among the wide variety of SPM techniques available [3], Kelvin probe force microscopy (KPFM) is an application of the atomic force microscope (AFM) for the evaluation of the surface potential with nanometric resolution. KPFM is a valuable investigative approach for the study of work functions via the measurement of the contact potential difference *V*_CPD_, that is, the difference between the electrostatic potential at the surface of the investigated structure and that of the KPFM probe [4].

KPFM has been extensively used in the PV field [5–7]; more specifically, by the analysis of interfaces in a solar device [8–9], it can reveal the presence of unintentional potential barriers or pn junctions, which hinder the extraction of the photogenerated charges. III–V-based solar devices belong to the PV technology of thin and ultrathin films in which layers with widths of the order of a few nanometres are often integrated for an optimal surface passivation or for better carrier extraction, considerably enhancing device efficiency [10–11]. Consequently, the experimental demonstration of the sensitivity of KPFM to the narrower layers can be crucial for the investigation and comprehension of local surface properties and charge transport mechanisms at interfaces [12].

Within this context, this work presents a study about the capability of cross-sectional KPFM for the study of a III–V multilayer stack under ambient conditions. In particular, we have investigated an InP/GaInAs(P) multilayer structure with layers of different widths and doping concentrations.

The first objective of this analysis is the evaluation of the spatial resolution of our KPFM setup under ambient conditions. The second objective is a full understanding of the *V*_CPD_ results combined with a description of the principal factors that affect KPFM measurements with the application of Kelvin probe (KP) numerical modelling. This enables the interpretation of the KPFM data, specifically to investigate the effect of space charge regions, surface defects, and illumination on *V*_CPD_ [13].

## Experimental

### Sample preparation

The structure of the studied sample is summarized in Table 1. This multilayer stack structure was epitaxially grown using a MOVPE process in an AIXTRON “Close Coupled Showerhead” reactor (6″ × 2″) at three different surface temperatures (580/600/640 °C). The n-type AXT substrate doping was typically in the range of 3 × 10^18^ to 5 × 10^18^ cm^−3^ with a thickness of 500 μm. Trimethylindium (TMIn), trimethylgallium (TMGa), phosphine (PH_3_), and arsine (AsH_3_) were the source materials, with hydrogen (H_2_) as a carrier gas. Diethylzinc (DEZn) was used as a source of Zn for p-type doping the InP:Zn and the phosphorus-based quaternary (GaInAsP:Zn) and GaInAs:Zn layers. The precursor flow was varied to cover a doping level range from 1 × 10^18^ cm^−3^ to 2.5 × 10^19^ cm^−3^. The first part of the structure was used to measure the growth rate of the non-intentionally doped InP layers (InP:nid) at surface temperatures of 600 and 640 °C. The reflectance signal, monitored with an in situ Laytec EpiCurve TT tool, did not show any difference between the growth rates at the two surface temperatures, which were around 2.13 μm·h^−1^*.* The second part of the structure corresponds to the Zn calibration stack used for the p-type cladding of the multiple quantum well-based structure. The doping concentration of the InP:Zn layers was varied from 2 × 10^18^ to 1 × 10^18^ cm^−3^. The three Zn doping levels of InP layers were purposely inverted along the growth direction to facilitate electrochemical capacitance–voltage (ECV) characterization due to the strong Zn diffusion. The InP:Zn and the GaInAsP:Zn layers were epitaxially grown at a surface temperature of 600 °C. Note that the GaInAsP:Zn layer is an intermediate layer with a doping concentration of 6 × 10^18^ cm^−3^ with the purpose to smooth the InP:Zn/GaInAs:Zn transition bandgap and to reduce contact resistances. Finally, a GaInAs:Zn contact layer was made at a lower temperature of 580 °C in order to reach a higher doping level around 2.5 × 10^19^ cm^−3^.

**Table 1:** Full structure of the investigated multilayer stack sample.

Layer	Material	Doping concentration (cm^−3^)	Thickness (nm)

substrate	InP:S	(3–5) × 10^18^	500 μm
buffer	InP	nid	100
interlayer	GaInAs	nid	5
buffer	InP	nid	300
interlayer	GaInAs	nid	5
buffer	InP	nid	250
cladding	InP:Zn	2 × 10^18^	500
cladding	InP:Zn	1.50 × 10^18^	750
cladding	InP:Zn	1 × 10^18^	500
transition	GaInAsP:Zn	6 × 10^18^	20
contact	GaInAs:Zn	2.50 × 10^19^	200

Before starting the KPFM analysis, the sample was cleaved, and a surface cleaning was carried out to expose a clean cross section. We performed a chemical treatment based on sequential ultrasonic baths of acetone, ethanol, and deionized water. The sample was then placed in 1% HF solution for 30 s to etch the top oxide layer. This step was followed by a rinsing with deionized water and drying in air. This procedure was necessary for an optimal KPFM analysis since the presence of a native oxide surface layer on top can lead to the measurement of a misleading *V*_CPD_ value [14].

### Kelvin probe force microscopy

**Figure 1:**
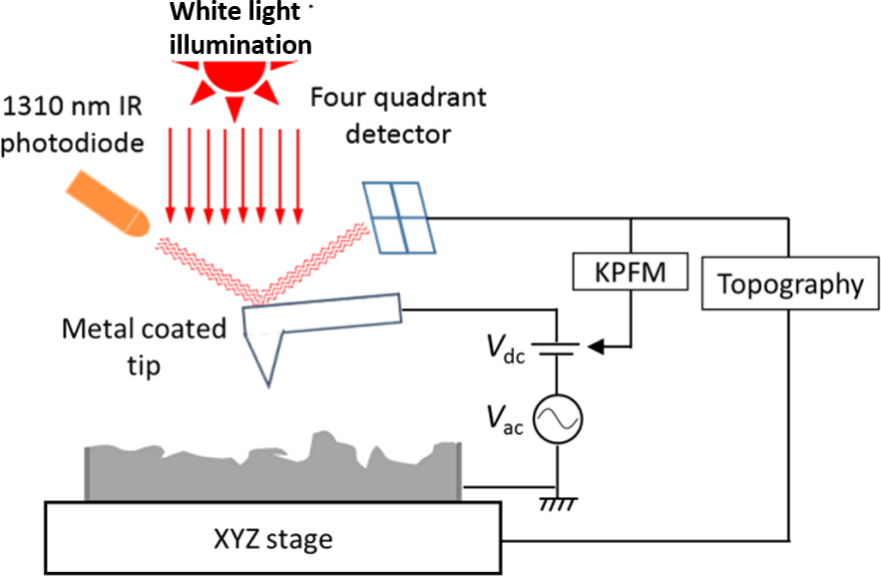
Schematic diagram of the KPFM system employed in this analysis. While an ac + dc potential is applied, the KPFM tip scans across a surface. The ac signal is sinusoidal with a frequency that equals the mechanical resonance of the cantilever. The four-quadrant detector gives feedback in order to minimize cantilever oscillation modifying the dc signal providing the sample surface potential relative to that of the tip. Figure 1 was reproduced from [13] (© 2019 C. Marchat et al., published by EDP Sciences, distributed under the terms of the Creative Commons Attribution 4.0 International License, https://creativecommons.org/licenses/by/4.0).

The following KPFM experimental procedures closely follow those described in [12]. KPFM evaluates the contact potential difference (*V*_CPD_) between the surface of metallic and semiconductive samples and a conductive AFM tip, which at equilibrium can be related to the work functions as:


[1]

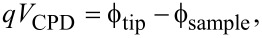



where ϕ_sample_ and ϕ_tip_ are the work functions of the sample and of the tip, respectively [4]. The *V*_CPD_ value is acquired by evaluating the DC voltage required to compensate for the electrostatic force generated between the tip and the sample, which, in turn, defines the KPFM signal [15]. KPFM was performed using a scanning probe microscopy system from AIST-NT (TRIOS platform) under ambient conditions and operated in the frequency-modulated KPFM (FM-KPFM) mode using a two-pass scanning mode, where the second pass was performed at a constant distance of 10 nm from the sample surface. Topographical data were collected on the first pass, whereas *V*_CPD_ was measured during the second one. The schematic of our KPFM setup is depicted in Figure 1.

The FM-KPFM mode was chosen over the amplitude-modulation mode (AM-KPFM) since it is well known that it provides better spatial resolution. In particular, in AM-KPFM the electrical force between the tip and the sample is directly evaluated, whereas in FM-KPFM the gradient of the force is analysed. As a result, FM-KPFM is more sensitive to local tip apex–sample surface interactions; therefore, long-range electrostatic interactions of the cantilever are reduced, as well as the effect of parasitic capacitances [16]. Additionally, in FM-KPFM, surface potential measurements are less dependent on the lift-height tip–sample distance than in AM-KPFM since this mode is less sensitive to static offsets induced by capacitive coupling or crosstalk [17].

The laser beam deflection system in our AFM employs a laser wavelength of 1310 nm, which is well below the bandgap of our sample; therefore, the parasitic laser absorption, which may interfere with the KPFM measurement, is reduced to negligible levels [13]. Highly doped n^+^-Si ARROW EFM tips (radius < 25 nm) with a conductive Pt/Ir coating at a resonance frequency of 75 kHz were used.

During KPFM measurements under ambient conditions, tip contamination is likely to occur because of pollutants that may be present on the sample surface causing a variation of ϕ_tip_ [18]. Hence, ϕ_tip_ was evaluated periodically in the course of the analysis using Equation 1 by measuring the *V*_CPD_ value of a freshly exfoliated surface of highly ordered pyrolytic graphite (HOPG) with ϕ_sample_ being equal to 4.6 eV [19]. The successively measured ϕ_tip_ values showed only small variations with values ranging between 5.65 and 5.75 eV.

KPFM measurements were performed under dark conditions and under illumination on the cross section of the sample. The acquisition of *V*_CPD/light_ enables the evaluation of the surface photovoltage (SPV), which is defined as the light-induced change of the contact potential difference at the surface of a photoactive material [20]. Since the surface potential of the tip is assumed to be unaffected by illumination, the difference between *V*_CPD/light_ and *V*_CPD/dark_ is equal to the change in surface potential of the sample between illumination and dark, which defines the surface photovoltage:


[2] $$\text{SPV}=V_{\text{CPD(light)}}-V_{\text{CPD(dark)}}.$$

It is important to mention that although KPFM is primarily a surface technique, the SPV can be sensitive to the presence of buried interfaces and/or deep charge trap states that may be present far from the surface in the bulk of semiconductors. Therefore, in our study the white light coming from the camera connected to the microscope was used. The white-light wavelength range is 400 to 700 nm, and for these wavelengths the penetration depth in GaInAs ranges between 10 and 100 nm. This makes our measurements mainly sensitive to the surface states and surface band bending. Additionally, a uniform illumination of the surface cross section was achieved thanks to the wide light spot.

Finally, the power density was 750 W·m^−2^ as measured by a thermal power sensor S401C from Thorlabs, which has a flat spectral response in the white-light range of wavelengths. This relatively low power density allows one to minimize the Dember effect since its contribution becomes significant only under high-injection conditions [20].

### KP modelling

In order to analyse the experimental characterization, scanning KP simulation was performed using the ATLAS software from Silvaco Inc. [21], controlled by the in-house software KELSCAN [13], which evaluates the contact potential and surface photovoltage as a function of the position.

The Silvaco ATLAS model solves the Poisson equation self-consistently coupled to carrier continuity and transport equations in the well-known drift diffusion model, which is given detail in [22] and not repeated here for brevity. The solution presented in this work assumes ohmic contacts and, therefore, Dirichlet boundary conditions fixing potential and carrier concentrations at the boundaries, as reported in section 3.5 of the SILVACO ATLAS manual. The ATLAS module solves semiconductor transport and continuity equations numerically in two dimensions and includes flexible descriptions of bulk and surface defect distributions. KELSCAN simulates the experimental setup by sequentially moving the AFM tip across the surface of the sample, statically solving the semiconductor equations at each position, and then evaluating the contact potential at each position from the field distribution calculated by ATLAS and exported to KELSCAN.

In order to replicate the experimental conditions, the radius of the tip is set at 25 nm, the distance between the tip and the sample cross section is set at 10 nm, and the ϕ_tip_ value is set at 5.7 eV, that is, the value measured on our tip as reported above. Note that KELSCAN allows one to simulate *V*_CPD_ measurements either under dark conditions or under illumination. In the case of “under illumination” simulations, the power density described in the above section was used.

KPFM is a surface technique; therefore, KPFM measurements are strongly influenced by the presence of surface defects. In order to provide a quantitative analysis of the experimental results, KELSCAN allows for the introduction of defects in a surface layer of arbitrary depth. The model of defects extending into the volume is physically more appropriate than a simpler two-dimensional surface distribution [23]. The introduced defect volume density of states (DOS), *N*(*E*) (eV^−1^·cm^−3^), is assumed to be homogeneous throughout the thickness of the defective layer, *t*_DL_, which we took equal to 1 nm. This can be translated into a surface density of states *N*_ss_(*E*) (eV^−1^·cm^−2^): *N*_ss_(*E*) = *N*(*E*) × *t*_DL_, with *t*_DL_ = 1 × 10^−7^ cm. In addition, the DOS consists of the sum of two distributions of monovalent donor and acceptor states, *N*_D_(*E*) and *N*_A_(*E*), respectively: *N*(*E*) = *N*_D_(*E*) + *N*_A_(*E*). These determine the charge neutrality level (CNL) of the surface defects. That is, when the Fermi level (*E*_F_) at the surface coincides with the CNL, there is no net charge coming from the surface defects. In contrast, when *E*_F_ is above (below) the CNL, surface defects are overall negatively (positively) charged. Here, to illustrate the effect of the defects on the band bending and on the measured surface potential profile, we introduced a simple constant DOS for both donor and acceptor states. The CNL is thus easily deduced from the ratio of the constant donor and acceptor DOSs. If they are chosen equal, the CNL is set at mid-gap, whereas it is moved towards the valence (conduction) band if the ratio of acceptor to donor DOS is larger (smaller) than 1 [24].

## Results

### KPFM cross-sectional investigation under dark conditions

**Figure 2:**
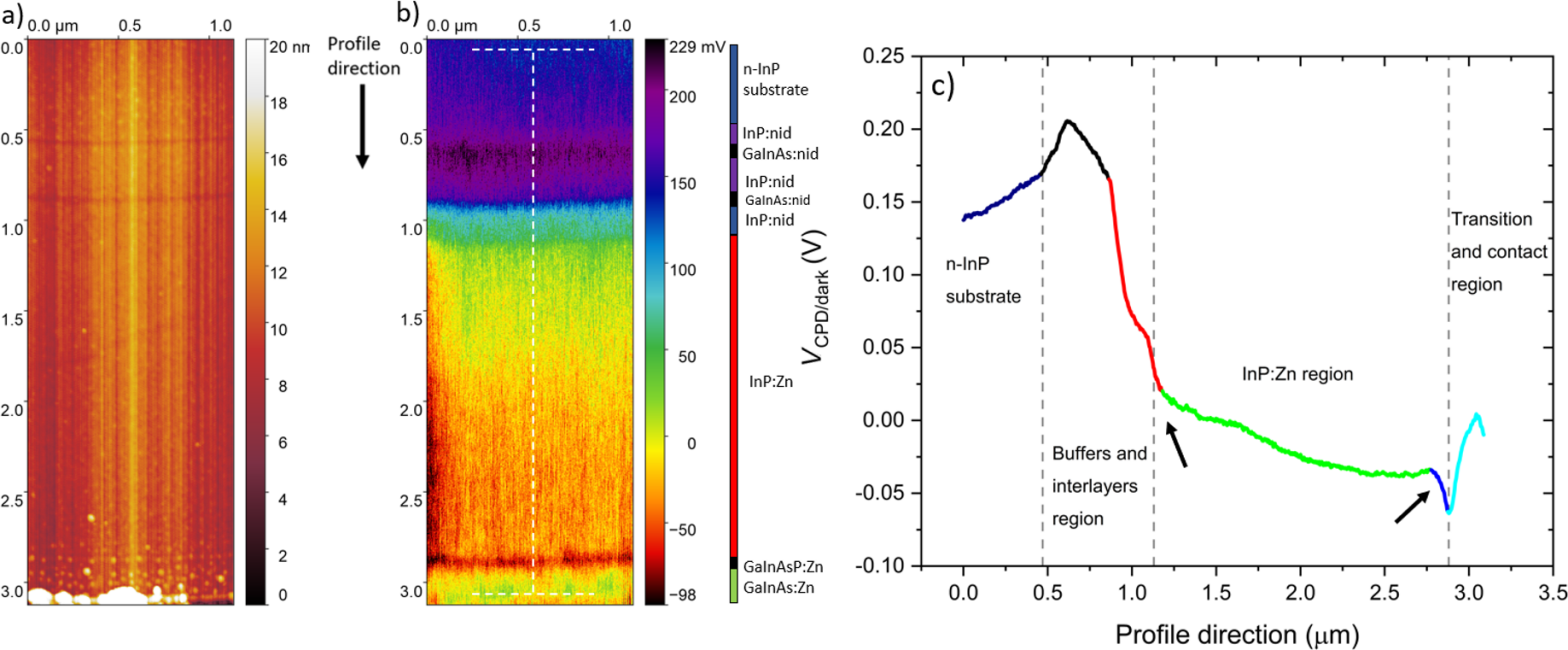
KPFM measurement under ambient conditions on the surface cross section of the sample under dark conditions: (a) topography and (b) *V*_CPD_ image. A vertical coloured bar is included to ease the identification of the different layers. The profile in (c) corresponds to the region identified by the dotted white segments in (b), each point of the profile (vertical) direction being an average of 207 points over a width of 0.7 μm along the *x* axis. Several regions along the structure have been highlighted using different colours (see text). Two black arrows indicate the space charge regions at the interfaces of the InP:Zn region.

The cross section of the sample was first investigated by KPFM under dark conditions, immediately after the chemical cleaning step. The topography and the associated *V*_CPD_ image are reported in Figure 2a and Figure 2b, respectively.

Note that the origin (0;0) is identified as a point in the InP substrate. Moving along the positive direction of the *y* axis, one will reach the end of the sample, that is, the surface of the 2D wafer (e.g., around 3.09 μm in Figure 2). In order to achieve a successful KPFM analysis, a low surface roughness is essential to obtain high-quality images since surface inhomogeneities can cause a topographical image imprint on the surface potential image. With sufficiently low surface roughness, the topographic influence on the measurement is minor, and the observed contrast of the *V*_CPD_ map is dominated by the surface potential such that topographic artefacts can be neglected.

A first look at the *V*_CPD_ image and the extrapolated profile (Figure 2c) allows for a qualitative analysis. KPFM successfully detects the n-InP substrate (from 0 to 0.46 μm), the InP:nid/GaInAs:nid region (from 0.46 to 1.12 μm), the InP:Zn region (from 1.12 to 2.87 μm), and the GaInAsP:Zn/GaInAs:Zn region (from 2.87 to 3.09 μm).

KPFM demonstrated a strong sensitivity on the local doping concentration as reported in a number of publications [12,25]. However, a clear identification of the 5 nm GaInAs:nid interlayers among the InP:nid buffer layers is not achieved in the *V*_CPD_ image. Nevertheless, their presence was still detected and represented in the *V*_CPD_ image by the dark and blue lines at 0.61 and 0.91 μm, respectively. The low resolution of the interlayers can be attributed either to their narrowness or to the experimental conditions since the two GaInAs:nid layers are well resolved in the topography image. Certainly, the width of these layers is narrower than the radius of the tip (<25 nm), and the operating conditions, namely the tip–surface distance and ambient measurements, negatively affect the resolution of KPFM measurements [26]. In particular, KPFM under ambient conditions is affected by the tip-averaging effect due to the long-range nature of the electrostatic force. The tip can sense multiple layers with different properties simultaneously, resulting in the detection of an averaged *V*_CPD_ at the interfaces [27].

During KPFM measurements, the tip scans the cross section from the n-InP substrate to the end of the sample; consequently, it will sense the surface potential variation along the structure. The progression of the *V*_CPD_ profile shows that four different slopes are present considering the region from the last InP:nid buffer layer to the GaInAs:Zn contact layer (from 0.86 to 3.09 μm). In particular, the first is located between the last InP:nid buffer layer and the first InP:Zn layer (from 0.86 to 1.17 μm), the second in the InP:Zn region (from 1.17 to 2.76 μm), the third between the last InP:Zn layer and the GaInAsP:Zn transition layer (from 2.76 to 2.87 μm), and the fourth between the GaInAsP:Zn transition layer and the GaInAs:Zn contact layer (from 2.87 to 3.09 μm). These regions have been identified with the colours red, green, blue, and light blue in the *V*_CPD_ profile, respectively.

The green profile represents the InP:Zn region; because of the comparable doping concentration of the three InP:Zn layers, a small variation of *V*_CPD_ of the order of 20 mV is expected to be measured along this region. However, the experimental *V*_CPD_ profile presents a *V*_CPD_ variation of the order of 50 mV along the InP:Zn region (from 1.13 to 2.87 μm). Several factors can influence KPFM measurements leading to this experimental evidence, namely the sample preparation, the experimental conditions, and the presence of surface defects. All these aspects have an impact on the surface potential, as we will see in the Discussion section (“KPFM experimental conditions and sample preparation”).

Regarding the other slopes pointed out above, their detection is attributable to the formation of space charge regions among the different layers along the structure [28]. Specifically, the *V*_CPD_ progression reflects the band bending present in the presence of depletion and accumulation regions. In particular, undoped InP crystals always contain different unintentional impurities due to the growth processes. The InP:nid layers fabricated at III–V Lab usually present an intrinsic n-type doping of the order of 10^15^ cm^−3^, which results in shallow donor energy levels within the energy gap. Since the intentional Zn p-type doping concentration is much greater than this residual n-type doping present in the InP:nid buffer, the space charge region is expected to be located almost exclusively in the buffer layer.

Similarly, two Zn doping concentration gradients are present from the last InP:Zn layer to the GaInAsP:Zn transition layer (from 1 × 10^18^ to 6 × 10^18^ cm^−3^) and from the GaInAsP:Zn transition layer to the GaInAs:Zn contact layer (from 6 × 10^18^ to 2 × 10^19^ cm^−3^). This results in two space charge regions situated almost completely in the InP:Zn layer and in the GaInAsP:Zn transition layer, respectively.

It is worth to mention that the band bending induced by the different space charge regions along the structure depends on the doping concentration (e.g., number of involved charge carriers) and on the width of the layers. Consequently, the corresponding *V*_CPD_ variation will depend on the same parameters [12].

**Figure 3:**
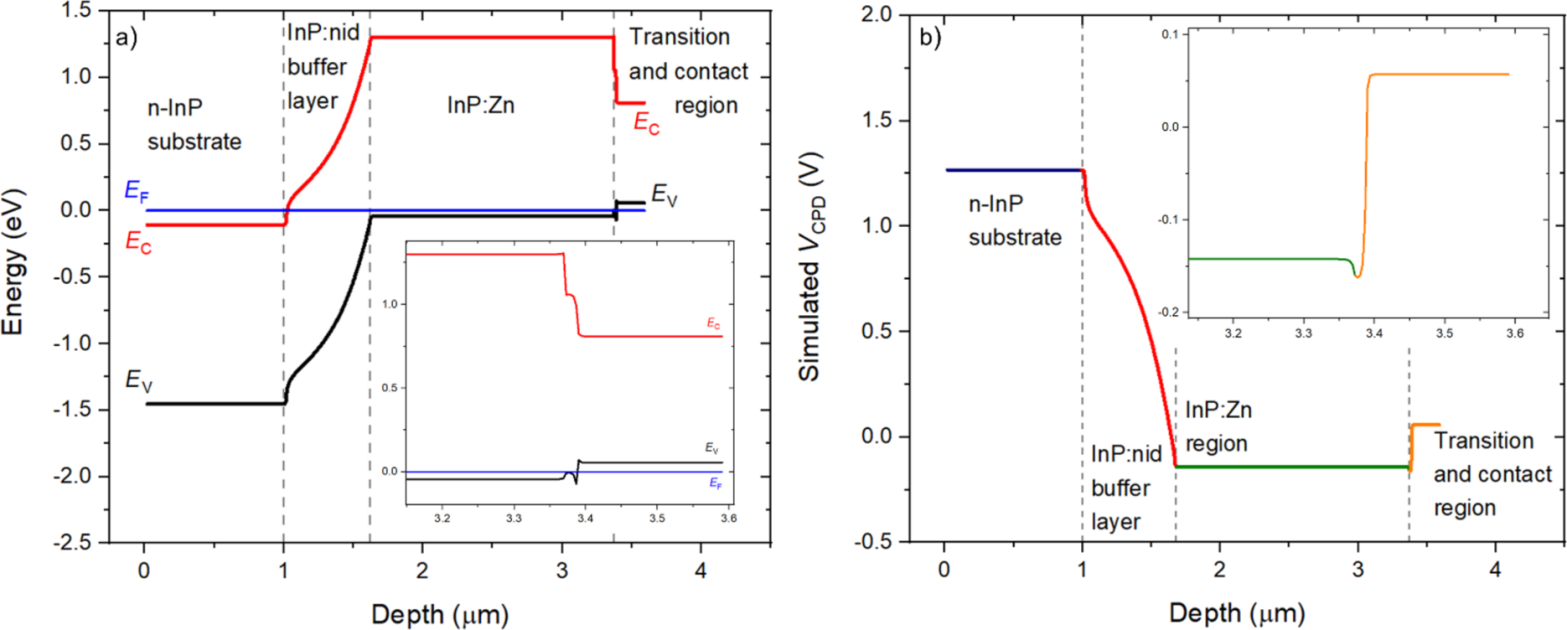
(a) Cross-sectional profile at equilibrium of the surface band energies (black: valence band maximum, *E*_V_, and red: conduction band minimum, *E*_C_) along the structure assumed free of any surface defects. The constant Fermi level, *E*_F_, is taken as the energy reference (blue horizontal line). (b) *V*_CPD_ evolution along the same simulated structure obtained by KP modelling. Note that both figures present an inset detailing the InP:Zn/GaInAsP:Zn/GaInAs:Zn interfaces close to the external surface of the sample.

In order to investigate the effect of the space charge on the measured *V*_CPD_, we have implemented theoretical modelling to this work. As a first step, we have simulated through ATLAS/Silvaco software [21] the energy bands profile of the analysed structure in the ideal case in which no surface defects are considered, qualitatively reproducing the expected energy bands profile in our sample. The widths and doping concentrations of the layers were chosen as reported in Table 1, whereas the other physical parameters (e.g., energy gaps) are present in the Silvaco database [21]. Note that in order to simulate the InP:Zn region, we have specified just one Zn doping concentration of 1.5 × 10^18^ cm^−3^. Furthermore, in order to replicate the experiment, we have included a metal layer on the left of the n-type InP substrate. Under these conditions, the metal layer represents the contact between the sample holder and the n-type InP substrate. Finally, the work function of the metal was set to be equal to that of the substrate to guarantee an ohmic contact. The simulated energy bands profile confirmed our hypothesis showing the induced band bending along three space charge regions at the InP:nid/InP:Zn, the InP:Zn/GaInAsP:Zn, and the GaInAsP:Zn/GaInAs:Zn interfaces, as shown in Figure 3a.

In particular, because of the low doping concentration of the InP:nid layer compared to the adjacent n-InP substrate and the InP:Zn region, a space charge extends over its complete width. The *V*_CPD_ profile across the InP:Zn/GaInAsP:Zn/GaInAs:Zn interfaces results from the different work functions. The work function of GaInAsP:Zn is slightly larger (by 0.04 eV) than that of InP:Zn, and it is substantially larger (by 0.22 eV) than that of the GaInAs:Zn contact layer, which leads to a decrease and an increase of potential, respectively. It is important to note that due to the narrowness of the GaInAsP:Zn transition layer (20 nm), the space charges at the two neighbouring heterojunctions overlap in this layer, leading to an asymmetric U-shape of the *V*_CPD_ profile. The asymmetric U-shape is also present in the experimental profile in Figure 2c (dark blue and light blue parts emphasizing the decrease and increase in potential, respectively). The mismatch of the conduction and valence bands between these materials then leads to the peculiar band energy diagram. Insets have been added to Figure 3a and Figure 3b to zoom in this region. Additionally, KP modelling [13] was used to simulate the *V*_CPD_ profile along the same structure assumed to be free of any surface defects for a quantitative evaluation of the effect of space charge on the surface potential (Figure 3b). *V*_CPD_ is proportional to the difference between the vacuum level and *E*_F_; therefore, the changes in the energy bands profile will be reflected in the simulated *V*_CPD_ profile.

The simulated *V*_CPD_ profile shows the same qualitative progression as the experimental profile reported in Figure 2c. However, several important differences can be noted by the comparison between the experimental and simulated *V*_CPD_. In particular, the experimental *V*_CPD_ profile (Figure 2c) seems to show that a part of the first space charge extends into the first InP:Zn layer (from 1.12 to 1.20 μm); similarly, the second space charge seems to extend more into the last InP:Zn layer than the modelling predicts (from 2.76 to 2.87 μm). These two regions are indicated by the two black arrows in Figure 2c. Additionally, the simulated *V*_CPD_ shows a flat profile a few nanometres inside the GaInAs:Zn contact layer, whereas a flat surface potential is not obtained experimentally. In other words, experimental surface potential variations occur over distances larger than one may expect solely from the extrinsic Debye lengths calculated from the nominal doping densities, which are only a few nanometres [29]. As a consequence, the lack of a sharp transition among the interfaces can cause difficulties in the identification of the position of the metallurgical junctions in the *V*_CPD_ image [28]. In particular, one reason can be found in the aforementioned tip-averaging effect: The tip still senses parts of the space charge in the InP:nid buffer layer and in the GaInAsP:Zn transition layer although being already on the first InP:Zn layer and on the GaInAs:Zn contact layer, respectively. Similarly, the tip starts to sense prematurely parts of the space charge inside the last InP:Zn layer. Furthermore, non-ideal abrupt junctions may contribute to this effect, for instance, because of dopant interdiffusion, as will be described in the following section.

Finally, the simulated *V*_CPD_ progression predicts an overall surface potential change of the order of around 1.34 V from the n-type InP substrate to the InP:Zn region. Conversely, this *V*_CPD_ variation in our experimental results is of the order of around 0.18 V. This is a first indication that the experimental surface potential is modified by the presence of surface states induced by surface defects since we know that KPFM is a surface technique and that the simulated *V*_CPD_ variation at this stage is based solely on bulk material properties and is not affected by any surface defects. Therefore, the experimental surface potential results to be less pronounced than in the “gedanken profile” that occurs far from the surface. This will be fully addressed in the Discussion section (“KPFM experimental conditions and sample preparation”).

### KPFM cross-sectional investigation under illumination

**Figure 4:**
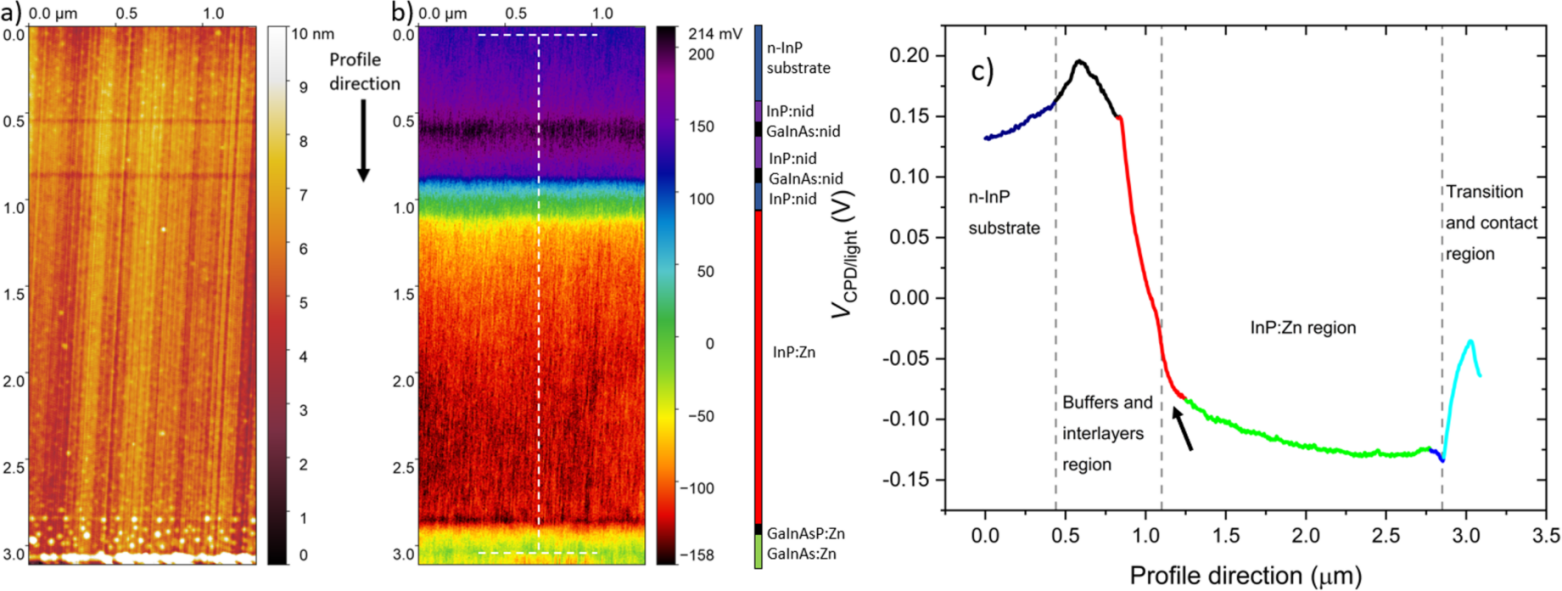
KPFM measurement under ambient conditions on the surface cross section of the sample under illumination: (a) topography and (b) *V*_CPD_ image. A vertical coloured bar is included to ease the identification of the different layers. The profile in (c) corresponds to the region identified by the dotted white segments in (b), each point of the profile (vertical) direction being an average of 207 points over a width of 0.7 μm along the *x* axis. Several regions along the structure have been highlighted using different colours (see text). The black arrow indicates the space charge region at the InP:nid/InP:Zn interface.

In order to study the effect of the illumination on the sample cross section, we have performed KPFM measurements under white-light illumination. The topography and the associated *V*_CPD_ image are reported in Figure 4a and Figure 4b, respectively. The *V*_CPD/light_ image of Figure 4b shows a significant contrast enhancement due to the interaction with the light compared to *V*_CPD/dark_ of Figure 2b. As a consequence, *V*_CPD/light_ results to be more homogenous all along the cross section than *V*_CPD/dark_, as shown in the corresponding *V*_CPD/light_ profile reported in Figure 4c. Moreover, the improvement of contrast also facilitates the identification of the narrower interlayers and of the position of the metallurgical junctions at the InP:nid/InP:Zn and the InP:Zn/GaInAsP:Zn interfaces, which were more undefined in the previous *V*_CPD/dark_ image.

Overall, the *V*_CPD/light_ profile follows the same evolution as the profile of *V*_CPD/dark_; also in this case, four different *V*_CPD_ slopes are present in the profile. In particular, the first is located between the last InP:nid buffer layer and the first InP:Zn layer (from 0.83 to 1.25 μm), the second in the InP:Zn region (from 1.25 to 2.78 μm), the third between the last InP:Zn layer and the GaInAsP:Zn transition layer (from 2.78 to 2.85 μm), and the fourth between the GaInAsP:Zn transition layer and the GaInAs:Zn contact layer (from 2.85 to 3.07 μm). These regions have again been identified with the colours red, green, blue, and light blue in the *V*_CPD_ profile, respectively.

Notably, the *V*_CPD/light_ profile along the InP:Zn region between 1.25 and 2.78 μm is flatter compared to that of *V*_CPD/dark_. This *V*_CPD/light_ profile is more consistent with what the modelling predicts for such small variations in the Zn doping concentration along the InP:Zn region. Conversely, at the beginning of the InP:Zn layer, from 1.10 to 1.25 μm (indicated by the black arrow), the *V*_CPD_ profile presents a steeper slope suggesting that the tip is still sensing the band bending induced by the space charge between the last InP:nid and the first InP:Zn layer. However, the tip-averaging effect alone cannot explain the detection of a space charge that extends for around 0.16 μm inside the first InP:Zn region. As a matter of fact, the diffusion of Zn impurities is likely to occur due to the high temperatures required for the growth of the material and the high diffusion coefficient of Zn in InP [30]. Therefore, the true spatial extent of the space charge region is not trivial to determine and may differ from what would be expected given the nominal structure of the sample. Conversely, the width of space charge region between the last InP:Zn layer and the GaInAsP:Zn transition layer is reduced and closer to the modelled one. Additionally, the detected surface potential change related to the space charge region at the GaInAsP:Zn/GaInAs:Zn interface is higher and closer to the simulation.

**Figure 5:**
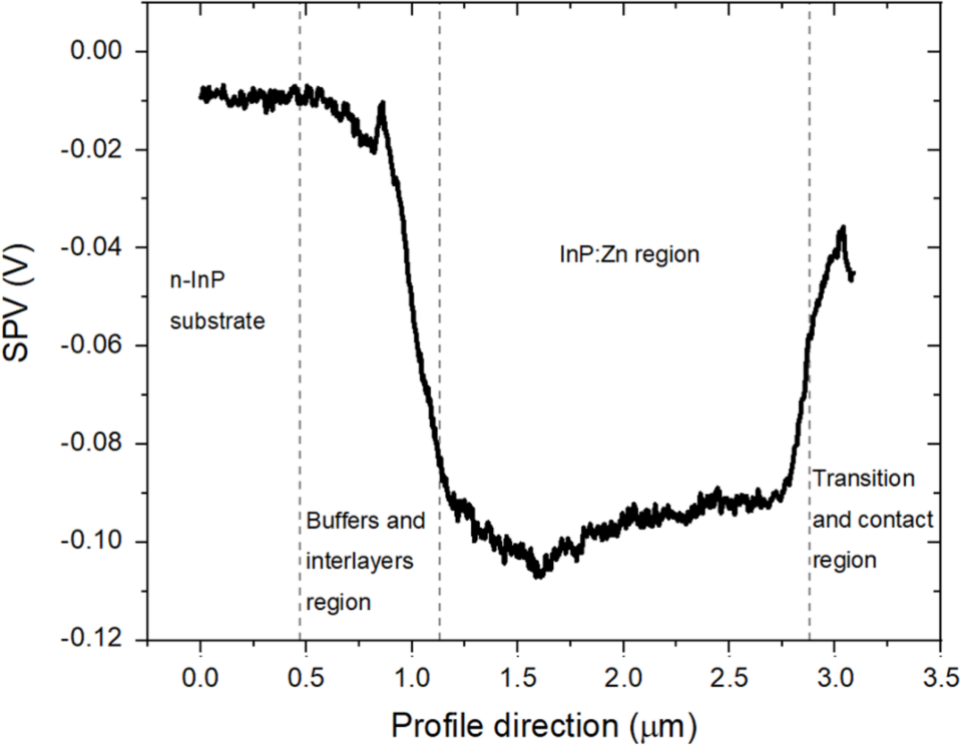
SPV profile along the structure calculated from the values of *V*_CPD/dark_ and *V*_CPD/light_ shown in the profiles of Figure 2c and Figure 4c, respectively, applying Equation 2.

As described in the Experimental section (“Kelvin probe force microscopy”), the SPV can be calculated by applying Equation 2 to the experimental values of *V*_CPD/light_ and *V*_CPD/dark_. The SPV along the structure is reported in Figure 5.

The SPV progression along the structure shows an overall negative SPV. For highly doped semiconductors in the absence of surface states (or for surface state densities small enough so that they cannot introduce significant surface band bending) a SPV signal close to zero is expected to be measured [20]. We therefore expect a vanishing SPV signal in the highly doped n-type InP substrate, which is degenerately doped at 5 × 10^18^ cm^–3^ with respect to the InP effective conduction band density of states (5.7 × 10^17^ cm^–3^ [31]). Experimentally the uncertainty on extracted SPV values can be evaluated at ±20 mV. Hence, the obtained value of around −10 mV in the highly doped n-type InP substrate is in good agreement with the theoretical expectation of vanishing SPV.

Furthermore, a negative SPV of around −95 mV is estimated for the InP:Zn region, which is consistent with the fact that a negative SPV is expected for a p-type semiconductor because of surface band bending due to surface states produced by surface defects. In particular, the detection of a negative SPV implies that a downward band bending is present in the vicinity of the surface [32]; this aspect will be fully addressed in the Discussion section (“Effect of the illumination on the VCPD”). Finally, it is worth mentioning that after illumination the initial conditions are restored, which excludes the presence of long-lived charge accumulation along the different junctions.

## Discussion

In this section the principal factors that affect KPFM measurements will be addressed in order to develop a methodology of analysis and apply it to experimental results.

### KPFM experimental conditions and sample preparation

Several factors can influence KPFM measurements, namely the experimental conditions and the status of sample surface and AFM tip. Additionally, as pointed out in the Results section (“KPFM cross-sectional investigation under dark conditions”), the presence of surface non-idealities (e.g., surface defects) has an effect on the surface potential and, thus, on the measured *V*_CPD_. All these aspects can lead to surface inhomogeneities, which result in *V*_CPD_ variations compared to an otherwise constant measurement on bulk material.

KPFM analysis was carried out under ambient conditions, which result in surface oxidation and in the adsorption of water molecules on the sample surface due to the humidity present in air [18]. Furthermore, a non-optimal deoxidation procedure may result in an inhomogeneous removal of the surface oxide. Additionally, the condition of the tip during the numerous scans along the sample cross section must also be considered. In particular, contamination of the tip is likely to occur due to pollutants (e.g., nano- and/or micrometre-size dust grains), which may be present on the sample surface leading to a variation of the tip surface potential.

The tip-averaging effect represents an important aspect of KPFM under ambient conditions, as revealed in the Results section (“KPFM cross-sectional investigation under dark conditions”). Even at extremely short tip–sample distances (5 nm), the tip-averaging effect can lower the lateral resolution as well as the measured KPFM signal [25]. This is especially evident in KPFM under ambient conditions, where typical tip–surface distances are of the order of tens of nanometres because of the vibrating tip amplitude necessary to achieve an acceptable signal-to-noise ratio.

**Figure 6:**
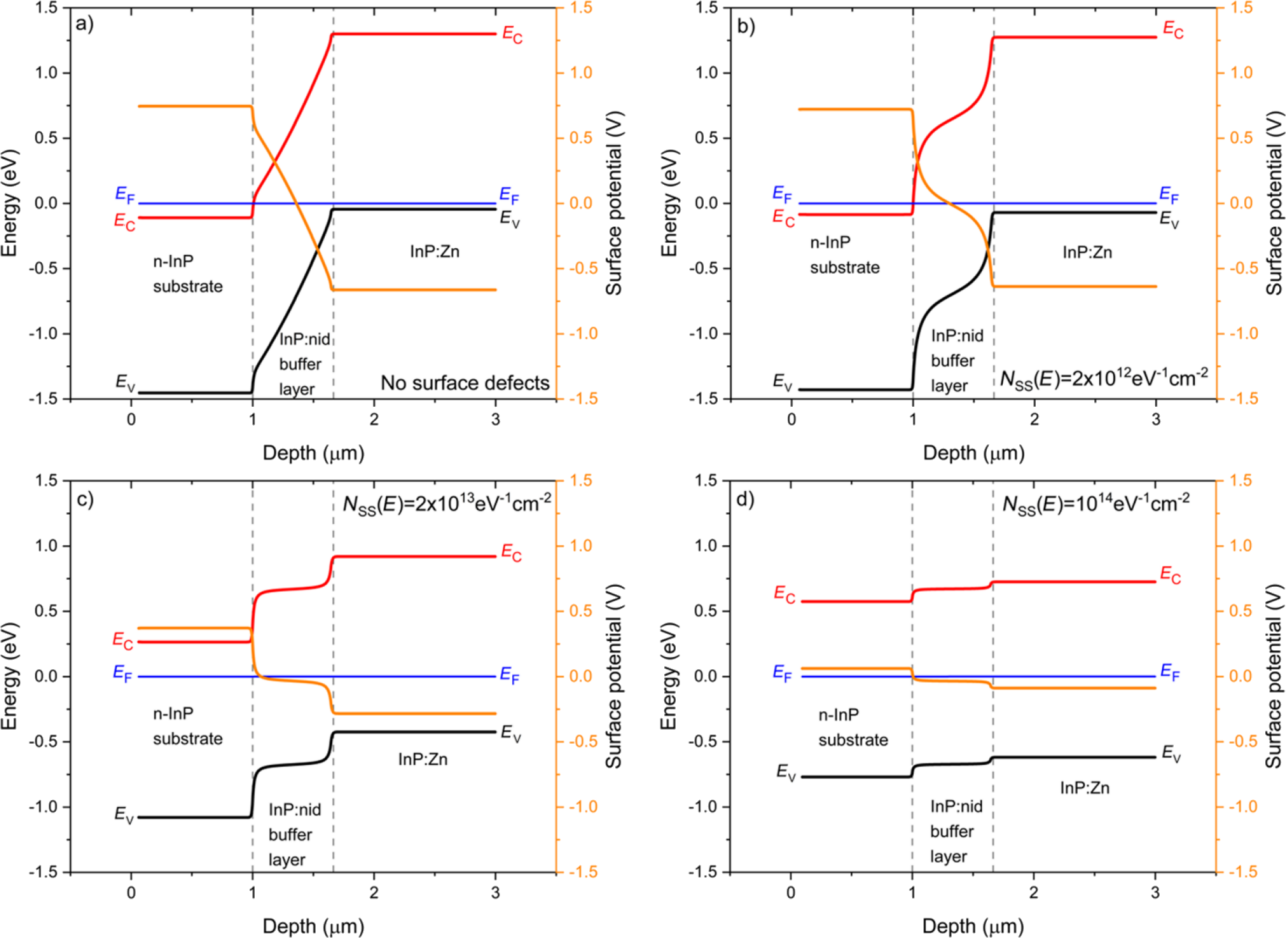
Cross-sectional profile at equilibrium of the surface band energies (black: valence band maximum, *E*_V_, and red: conduction band minimum, *E*_C_) along a simulated n-InP/InP:nid/InP:Zn structure considering surface defects densities made of the sum of constant and identical acceptor-like and donor-like defect distributions (in eV^−1^·cm^−2^): (a) 0, (b) 2 × 10^12^, (c) 2 × 10^13^, and (d) 1 × 10^14^. The energy reference is taken at the constant Fermi level, *E*_F_ (blue horizontal line). The profile of the surface potential is also shown in orange (right *y* axis of the graphs).

Finally, it is well documented in the literature [20] that the cleavage procedure produces surface defects, which strongly impact the *V*_CPD_. In order to study the effects of surface defects on the *V*_CPD_, we have extended the energy band simulations to a non-ideal case in which constant distributions of acceptor-like and donor-like defects have been introduced at the surface, as described in the Experimental section (“KP modelling”). In order to clarify the analysis and focus on essentials, we have simulated a simpler structure with respect to the analysed multilayer sample, in which we did not include the 5 nm InGaAs:nid interlayers and the final InGaAs(P):Zn transition and contact layers. Specifically, we have compared the ideal structure free of surface defects to three different cases in which identical acceptor-like and donor-like surface defects densities of 1 × 10^12^, 1 × 10^13^, and 5 × 10^13^ eV^−1^·cm^−2^ (taken to be constant throughout the bandgap) were introduced at the surface. The results are reported in Figure 6. In this specific case, the charge neutrality level of surface defects is set at mid-gap. Thus, increasing the surface defect densities will produce a pinning of the Fermi level at the neutrality level of the surface states and the energy of valence and conduction bands will appear symmetric with respect to mid-gap position [24]. In particular, it is possible to observe this trend even at relatively low surface defects densities (2 × 10^12^ eV^−1^·cm^−2^, see Figure 6b) in the InP:nid layer because of the low doping concentration (1 × 10^15^ cm^−3^) compared to the other two layers. Conversely, in the n-InP substrate and in the InP:Zn layer, this trend is only well evidenced when high surface defects densities (above 10^13^ eV^-1^·cm^−2^) are introduced at the sample surface; the trend is already visible for 2 × 10^13^ eV^−1^·cm^−2^ and really clear for 1 × 10^14^ eV^−1^·cm^−2^ in Figure 6c and Figure 6d, respectively.

Increasing the surface defect densities leads to an increase of the valence and conduction band energies within the n-InP substrate and to a decrease in the InP:Zn layer, so that the overall potential drop across the junctions is significantly reduced, from 1.42 V in Figure 6a to 0.15 V in Figure 6d. Specifically, to an increase of energy corresponds a decrease of surface potential, which reflects the upward band bending induced by the presence of surface defects. Conversely, a decrease of energy corresponds to an increase of surface potential, which reflects the downward band bending induced by the presence of surface defects.

We conclude that the presence of surface defects can explain the overall experimental *V*_CPD_ variation along the structure that is less pronounced than in the simulated ideal case of a defect-free surface, as described in the Results section (“KPFM cross-sectional investigation under dark conditions”). This conclusion on the overall mitigation of the *V*_CPD_ variation is not changed if we choose other surface defect density distributions (not constant vs energy) that produce different charge neutrality levels in the energy gap (which is not presented here for brevity).

**Figure 7:**
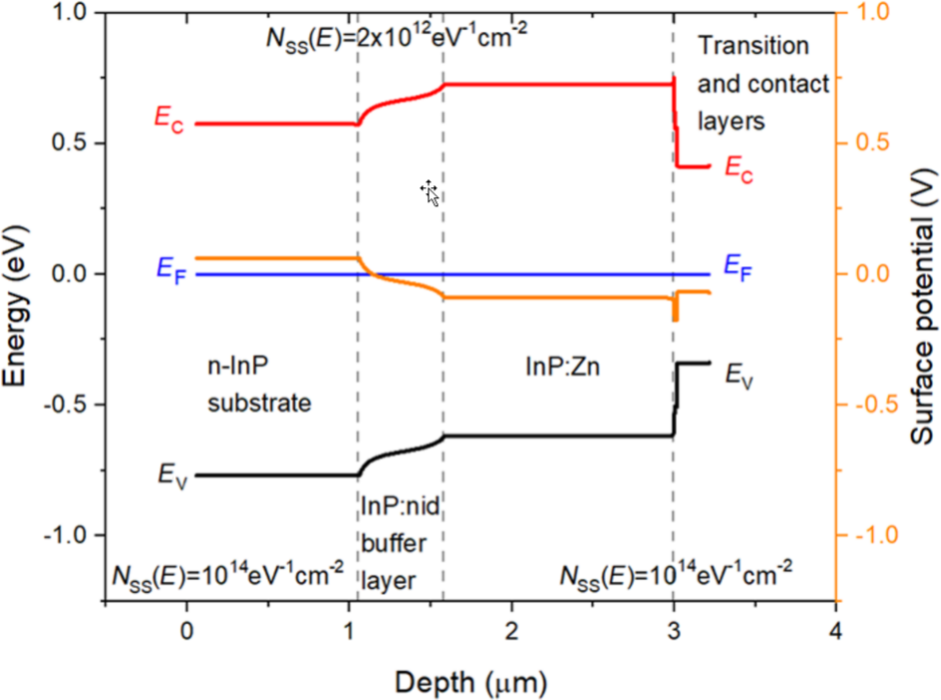
Cross-sectional profile at equilibrium of the surface band energies (black: valence band maximum, *E*_V_, and red: conduction band minimum, *E*_C_) along a simulated n-InP/InP:nid/InP:Zn/GaInAsP:Zn/GaInAs:Zn structure considering different surface defect densities in the various layers. For the n-InP substrate and the InP:Zn, GaInAsP:Zn, and GaInAs:Zn layers a total surface defect density (made of the sum of constant and identical acceptor-like and donor-like defect distributions) of 1 × 10^14^ eV^−1^·cm^−2^ was considered, whereas for the InP:nid layer a total surface defect density of 2 × 10^12^ eV^−1^·cm^−2^ was introduced. The energy reference is taken at the constant Fermi level, *E*_F_ (blue line). The profile of the surface potential is also shown in orange (right *y* axis).

However, large surface defect densities not only mitigate the overall change in *V*_CPD_, but they are also responsible for strong changes in the shape of the surface potential. For instance, in Figure 6d the surface potential appears flat along the simulated structure with the exception of very narrow transition regions at the two layer interfaces. In other words, large surface defect densities also decrease the effective screening lengths compared to the ones calculated exclusively from the nominal doping densities, due to the extra charges directly provided by the surface states. The essentially constant flat profile in the InP:nid buffer layer strongly departs from the progressively decreasing profile observed experimentally in Figure 2c. In order to provide an explanation for the observed experimental profile that is both mitigated and progressively decreased in this buffer layer, it is necessary to decrease the surface defect density in the buffer layer, while keeping a very large value in the external n-InP substrate and p-InP:Zn layer. Therefore, the n-InP substrate and the p-InP:Zn layer require a high value of *N*_SS_ = 1 × 10^14^ eV^−1^·cm^−2^, whereas the InP:nid layer requires a lower value of *N*_SS_ = 2 × 10^12^ eV^−1^·cm^−2^. Furthermore, in order to provide a more quantitative explanation of the experimental profile of Figure 2c, the GaInAsP:Zn transition and GaInAs:Zn contact layers have been included again in the simulated structure (*N*_SS_ = 1 × 10^14^ eV^−1^·cm^−2^). The energy bands and surface potential profiles simulated with these parameters are shown in Figure 7.

The surface potential shown in Figure 7 is in good agreement with the experimental profile of Figure 2c. In particular, the potential drop from the n-InP substrate to the InP:Zn layer is comparable to the 0.18 V obtained experimentally. Additionally, the shape of the surface potential in the InP:nid layer shows a progressive change extending all over the InP:nid buffer layer. Finally, the GaInAsP:Zn transition and GaInAs:Zn contact layers are again consistent with the higher value of *N*_SS_ = 1 × 10^14^ eV^−1^·cm^−2^. In particular, the potential difference between the InP:Zn and the GaInAs:Zn contact layers is also attenuated with respect to the ideal case shown in Figure 3b as in the experimental *V*_CPD_ profile of Figure 2c. Overall, this approach demonstrates that the surface defect density variations provide good agreement with the experimental surface potential profile of Figure 2c.

In conclusion, a quantitative description of the accurate surface defects distributions that characterize the surface of semiconductors materials is a complex task as it is not always certain that surface defects are homogeneously distributed across the entire cross section. This is particularly true in our case since the several layers present different physical properties because of varying doping types and concentrations [33].

In order to overcome these challenges related to the operating conditions and to the cleaving process presented in this paragraph, KPFM measurements can be performed in ultrahigh vacuum (UHV) at an optimal surface–tip distance of the order of a few nanometres [34] with particular attention to the sample preparation either in the deoxidation and cleaving process.

### Effect of the illumination on the *V*_CPD_

In the Results section (“KPFM cross-sectional investigation under illumination”), we have pointed out the enhancement of contrast in the *V*_CPD_ image under white-light illumination of the sample cross section. In particular, since the bulk lattice periodicity is interrupted at the surface of a cleaved semiconductor, surface reconstruction and formation of dangling bonds of surface atoms may occur, creating surface states within the energy bandgap. For instance, these surface states can pin the Fermi level and cause downward (upward) band bending from the bulk to the surface in a p-type (n-type) semiconductor in the case of the formation of a depletion (or inversion) space charge layer imposed by the charge neutrality condition [32,35].

**Figure 8:**
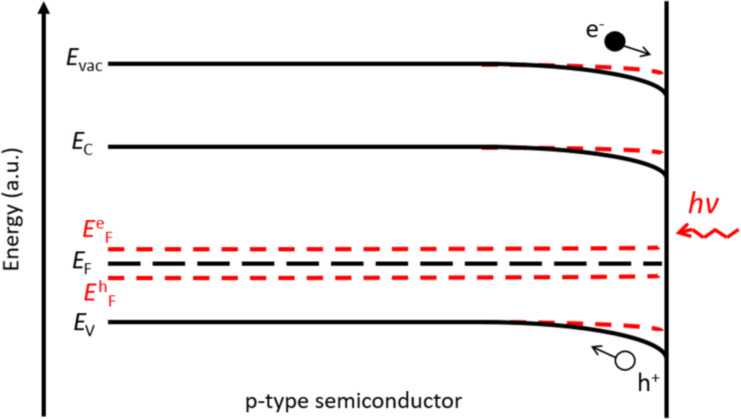
Representation of the energy band profile in a p-type semiconductor under dark conditions and under illumination depicted by black solid lines and red dashed lines, respectively. *E*^e^_F_ and *E*^h^_F_ represent possible profiles for the quasi-Fermi levels for electrons and holes, respectively.

By illuminating the sample, a SPV is generated by the drift and diffusion of photogenerated carriers towards the surface, which counteracts the defect-induced band bending energy variations [20]. As illustrated in Figure 8, in the case of downward surface band bending in an p-type semiconductor, photogenerated holes are repelled from the surface, while photogenerated electrons flow in the direction of the surface, balancing the positive charges corresponding to empty donor-type surface states. This results in a reduction of surface band bending and a decrease of surface potential, that is, a negative SPV.

Conversely, in the case of upward surface band bending in an n-type semiconductor, photogenerated electrons are repelled from the surface, while photogenerated holes flow towards the surface, balancing the negative charges corresponding to ionized occupied acceptor-type surface states, that is, a positive SPV.

As shown in Figure 5, an overall negative SPV was calculated along the structure, and a SPV of −95 mV was obtained in the InP:Zn region, which seems in good agreement with the expected trend in a p-type layer with surface defects. However, in case of pn junctions, the SPV can also include the contribution of the open-circuit voltage (*V*_OC_) of the pn junction due to the splitting of the quasi-Fermi levels of electrons and holes and the related charge separation at the junction. In our case, because the n-type side of the junction (substrate) is grounded, we expect a positive SPV contribution from the *V*_OC_ of the pn junction at the surface of the p layer outside the space charge region of the pn junction. Therefore, the SPV measured in the InP:Zn region should be a trade-off between the negative contribution due to the flattening of surface defect-related band bending and the positive contribution of *V*_OC_. As a consequence, the slightly negative SPV value of −95 mV measured in the InP:Zn region indicates a weaker contribution of the pn junction (*V*_OC_) compared to the change in surface band bending related to surface defects.

In order to provide a quantitative analysis of this experimental evidence, we have calculated the conduction and valence band energy shift induced by the illumination simulating two simple structures. The first one is metal/n-InP/air, the n-type InP simulates our n-type substrate with a doping concentration of 5 × 10^18^ cm^−3^, and the second one is metal/InP:Zn/air, with InP:Zn having a p-type doping concentration of 1.5 × 10^18^ cm^−3^, similarly to the p-doped layer in our sample. In these simulations, the back metal/InP contact was assumed to be ohmic in both structures. We introduced unequal donor-like and acceptor-like surface defect densities. Specifically, the donor-like defect density was chosen equal to 1 × 10^13^ eV^−1^·cm^−2^, and the acceptor-like was 20 times lower, 5 × 10^11^ eV^−1^·cm^−2^, resulting in a charge neutrality level very close to the conduction band of InP.

Under these conditions, a SPV close to zero and a negative SPV are expected for the n-type InP substrate and for the InP:Zn layer, respectively. The simulated results gave a SPV close to zero (very slightly positive) and a negative SPV of −356 mV for the n-type InP substrate and for the InP:Zn layer, respectively.

In this specific case, a *V*_OC_ of 261 mV would have been produced across the pn junction considering the experimental SPV result of −95 mV. This surprisingly low *V*_OC_ value could be explained either by a poor material quality of the sample, in which a high density of bulk defects is responsible for reducing the carrier lifetime, or by the lack of a true ohmic contact between the sample holder and the n-type InP substrate during the KPFM measurement. In the second case, a potential barrier would be present at the metal contact/n-InP substrate interface, which could reduce the overall *V*_OC_. Nevertheless, these considerations need further investigations and further support from modelling.

## Conclusion

In this contribution, it is shown that KPFM under ambient conditions is a valuable tool to investigate III–V multilayer stacks with high spatial resolution of down to 20 nm. The verified sensitivity of our KPFM setup to the narrower layers will be crucial for the study of the cross sections of operating solar device in future works.

The analysis of the surface potential profile identified the presence of space charge regions and, thus, the formation of several junctions along the stack. The complexity of the analysed structure combined with the ambient operating conditions caused challenges in the identification of the real position of the junctions in the *V*_CPD_ image.

KPFM measurements are significantly affected by surface defects and other surface inhomogeneities. In particular, numerical modelling and analysis indicated that surface defects are responsible for a significant departure of the magnitude of the surface potential from the value in the bulk material. Also, we showed that the observed potential profile along the cleaved surface of the n-InP/InP:nid/p-InP:Zn heterojunction stack can be explained by large surface defect densities in the highly doped n-InP and p-InP:Zn layers, with a much lower defect density in the InP:nid buffer layer.

With further characterization and analysis, we have shown that white-light illumination reduces the surface band bending induced by surface defects, providing an enhancement of the contrast in the *V*_CPD_ image. The analysis of the SPV variation along the structure cross section further suggests that either bulk defects or a non-ohmic contact between the metallic sample holder and the n-type InP substrate may exist. For future work, it will be necessary to assure a good ohmic contact between the sample holder and the sample and to carry out complementary characterization of the optoelectronic properties of the layers to refine the analysis of the results.
